# A public survey of traditional, complementary and integrative medicine use during the COVID-19 outbreak in Hong Kong

**DOI:** 10.1371/journal.pone.0253890

**Published:** 2021-07-01

**Authors:** Chun Sing Lam, Ho Kee Koon, Vincent Chi-Ho Chung, Yin Ting Cheung

**Affiliations:** 1 School of Pharmacy, Faculty of Medicine, The Chinese University of Hong Kong, Hong Kong, Hong Kong; 2 School of Chinese Medicine, Faculty of Medicine, The Chinese University of Hong Kong, Hong Kong, Hong Kong; 3 Jockey Club School of Public Health and Primary Care, Faculty of Medicine, The Chinese University of Hong Kong, Hong Kong, Hong Kong; Endeavour College of Natural Health, AUSTRALIA

## Abstract

**Background:**

During COVID-19, the public actively sought non-pharmacological and self-management approaches to prevent infection. Little is known on the use of traditional, complementary and integrative medicine (TCIM) by the public as preventive measures. This study investigated the prevalence and patterns of TCIM use during the pandemic, and identified factors associated with its use among the general population in Hong Kong.

**Methods:**

An online cross-sectional survey was conducted from November to December 2020. The survey solicited information on the respondents’ sociodemographic characteristics, risk perception of the pandemic, and use of TCIM before and during the pandemic. Logistic regression analysis was conducted to determine predictors of TCIM use.

**Results:**

In total, 632 responses (completion rate = 88.1%) were analyzed. TCIM was used by 44.0% of respondents during the pandemic. The most popular forms of TCIM were vitamins or other dietary supplements (n = 160, 25.3%) and Chinese herbal medicine (n = 122, 19.3%) during the pandemic. The most frequently reported indication was strengthening the immune system, especially for vitamins or other dietary supplements (n = 142/160, 88.8%). Respondents who reported using TCIM were more likely to be female (adjusted odds ratio [aOR] = 1.82, 95% confidence interval [CI] = 1.29–2.59), had higher education attainment (aOR = 2.21, 95% CI = 1.39–3.59), and older-aged (age >55 years: aOR = 1.77, 95% CI = 1.04–3.02). Respondents who resided in districts with moderate to high number of confirmed COVID-19 cases (aOR = 1.60, 95% CI = 1.07–2.42) and had a higher level of risk perception (aOR = 1.04, 95% CI = 1.01–1.07) were also more likely to use TCIM.

**Conclusion:**

TCIM was used commonly in Hong Kong during the COVID-19 pandemic. While vaccination and social distancing remain the mainstay of controlling the pandemic, professional bodies should proactively consider public preferences and provide information regarding the effectiveness and safety of TCIM for COVID-19 prevention and treatment.

## Introduction

The World Health Organization (WHO) declared coronavirus disease 2019 (COVID-19) as a Public Health Emergency of International Concern on January 30, 2020 [1], and later as a pandemic on March 11, 2020. Despite continuous efforts to develop effective treatments and vaccines, the spread of the disease remains unchecked. As of February 14, 2021, more than 100 million cases of COVID-19 and more than 2 million related deaths have been reported worldwide [2]. In Hong Kong, the first case of COVID-19 was reported on January 23, 2020. As of February 14, 2021 [3], more than 10,000 COVID cases and 187 related deaths had been reported in Hong Kong.

Considerable interest and attention has been given to traditional, complementary and integrative medicine (TCIM) over past few decades, and a growing body of evidence indicates the usefulness of such approaches in combating emerging infectious diseases [4, 5]. According to the WHO, traditional medicine is “the sum total of the knowledge, skill, and practices based on the theories, beliefs, and experiences indigenous to different cultures, whether explicable or not, used in the maintenance of health as well as in the prevention, diagnosis, improvement or treatment of physical and mental illness,” while complementary medicine refers to “a broad set of health care practices that are not part of that country’s own tradition or conventional medicine and are not fully integrated into the dominant health-care system” [6]. The scope of TCIM includes natural products, mind-body practices and other complementary approaches such as traditional medicines [7]. Several reviews have evaluated the potential benefits of TCIM use, particularly traditional Chinese medicine, vitamins and herbal medicine, in the treatment of COVID-19 [8]. Furthermore, meta-analyses have shown the effectiveness of Chinese herbal medicine in improving the treatment outcomes and reducing the symptoms such as fever and fatigue of COVID-19 patients [9–11].

To date, WHO and the local health authority have emphasized the non-pharmacological interventions, such as maintaining good hygiene and reducing contact that could facilitate viral transfer, to combat COVID-19 [12, 13]. However, extended periods of home confinement and social distancing during the COVID-19 pandemic have had adverse effects on people’s physical and mental health, as evidenced by increases in the prevalence of mental health disorders and the frequency of negative lifestyle changes [14, 15]. TCIM may help to restore a person’s quality of life, enable self-care and promote health. Several TCIM approaches, including herbs, vitamins, aromatherapy and mind-body practices, have been proven useful in reducing stress and anxiety and improving immunity, and therefore could potentially enhance physical and mental resilience during the COVID-19 pandemic [16].

Previous studies have investigated the use of TCIM among patients with an active infection or after recovery from an infection [17–20], including one Indian study on isolated COVID-19 patients [17]. However, few have explored TCIM usage as a preventive measure among the public during an epidemic. One study found that 76.1% of the respondents at a community hospital in South Korea used one or more types of TCIM during the 2015 Middle East respiratory syndrome (MERS) outbreak [21]; in another study, 22.1% of participants reported using supplements during a COVID-19 outbreak in Saudi Arabia [22]. To our knowledge, no comprehensive studies have explored the prevalence of TCIM use (both oral and non-oral) and the associated predictors in a Chinese population during a pandemic or compared the patterns of use before and during an outbreak. Therefore, the aims of this survey-based study were to 1) explore the prevalence and patterns of TCIM use among Hong Kong residents before and during the COVID-19 pandemic and 2) identify the predictors of TCIM use in this population.

## Methods

### Study design and setting

This cross-sectional survey study was conducted over a 6-week period from November 2 to December 18, 2020. The study was approved by the Survey and Behavioural Research Ethics Committee of the Chinese University of Hong Kong (Reference no. SBRE-20–101), and the study has been carried out in accordance with the Declaration of Helsinki. The study procedures and results are reported according to the Checklist for Reporting Results of Internet E-Surveys (CHERRIES) [23] (S1 Table in S1 File).

### Participants

The link of the survey was posted on publicly accessible social media platforms hosted by an academic institution. Eligible participants were invited to respond to the survey via social media platforms and they were encouraged to forward the link to other potential participants. We included participants who self-identified as Hong Kong residents, were at least 18 years of age and could comprehend written traditional Chinese.

### Data collection

The questionnaire was run and managed using Qualtrics XM (Qualtrics, Provo, UT), an online survey development platform, and was reviewed by two researchers to assess the appropriateness and clarity of the questions. Pilot testing of the questionnaire was conducted on ten individuals to ensure feasibility and readability in the targeted population. At the beginning of the survey, the study objectives were explained, and the participants were asked to provide informed consent before proceeding to the questions.

The survey comprised 30 questions categorized into three major sections (S2 Table in S1 File). The questions in the first section solicited the sociodemographic characteristics of the respondents. The second section included questions about the respondents’ COVID-19 status and risk perception. A 11-point scale (0 = not at all, 10 = very much) was used to assess the respondents’ levels of concerns. The third section addressed the respondents’ TCIM use one year before and during the pandemic. The onset of the pandemic was defined as December 2019 when the first outbreak in China was announced [24]. Respondents were asked to indicate the sources of information regarding TCIM and their reasons for using a particular type of TCIM.

### Data analysis

The statistical analyses were conducted using R, version 4.0.3. The respondents’ sociodemographic characteristics, health status, TCIM usage frequency and pattern and the reported indications are summarized using descriptive statistics. The prevalence of TCIM use one year before the pandemic and during the pandemic were compared using the McNemar test. The risk perceptions of COVID-19 among TCIM users and non-users were compared using the Mann–Whitney U test.

Univariate and multivariable logistic regression analyses were used to determine the predictors of TCIM use during the COVID-19 pandemic. The potential predictors included demographics (age, gender, religion), socioeconomic factors (education level, employment status, income level, residential area), clinical factors (chronic diseases, medication history, prior TCIM use) and risk perception (severity of outbreak in the residential area from January to October 2020, reported level of concerns) [25, 26]. For TCIM modalities used by more than 10% of the overall sample and at least 100 respondents during the pandemic, subgroup analyses were conducted to explore the factors associated with their uses. For all of the analyses, a p-value of < 0.05 was considered statistically significant. Variation inflation factor (VIF) was computed to detect any multicollinearity in the regression models. A VIF of 2.5 and higher is suggestion of potential multicollinearity [27].

## Results

### Characteristics of the respondents

There were 632 valid responses (n = 632/717, response rate:88.1%) included in the final analysis. The socio-demographic characteristics summary was shown in Table 1. The majority of the participants were female (n = 399, 63.1%) and over half of them were aged above 35 (n = 361, 57.1%). They were mostly employed (n = 462, 73.1%), and had attained an education level above secondary school (n = 516, 81.6%). Most respondents had either received a negative COVID-19 test result (n = 252, 39.9%) or had not been tested and were not suspected to have been infected (n = 370, 58.5%).

**Table 1:** Characteristics of TCIM user and non-user during COVID-19 (n = 632).

	N (%)
**Gender**	
Male	233 (36.9)
Female	399 (63.1)
**Age**	
18 to 35	271 (42.9)
>35 to 55	234 (37.0)
>55	127 (20.1)
**Religion**	
Yes	252 (39.9)
No	380 (60.1)
**Education level**	
Secondary school or below	116 (18.4)
Primary school or below	8 (1.3)
Secondary school	108 (17.1)
Higher diploma, degree or above	516 (81.6)
Higher diploma	78 (12.3)
Bachelor	186 (29.4)
Master or above	236 (37.4)
Other higher education	16 (2.5)
**Employment status**	
Employed	462 (73.1)
Housewives/unemployed/retired	118 (18.7)
Unemployed	14 (2.2)
Housewives	38 (6.0)
Retired	66 (10.5)
Students^a^	52 (8.2)
**Family income**	
≤$10000	78 (12.3)
>$10000	554 (87.7)
**District resided in (by median household income)**^b^	
High-income districts	158 (25.0)
Middle-income districts	251 (39.7)
Low-income district	223 (35.3)
**Chronic illnesses**	
Yes	171 (27.1)
Cardiovascular	79 (12.5)
Musculoskeletal	56 (8.9)
Diabetes	29 (4.6)
Cancer	26 (4.1)
Respiratory	17 (2.7)
Gout	8 (1.2)
Others	46 (7.3)
No	461 (72.9)
**Chronic medication**	
Yes	86 (13.6)
No	85 (13.4)
**History of using TCIM**	
Yes	306 (48.4)
No	326 (51.6)
**Respondents’ COVID status**	
COVID-positive and currently on treatment	2 (0.3)
COVID-positive and have recovered	0 (0)
COVID-negative	252 (39.9)
Not tested but suspect to have been infected	8 (1.3)
Not tested and not suspect to have been infected	370 (58.5)
**Family members’ COVID status**	
COVID-positive and currently on treatment	0 (0)
COVID-positive and have recovered	2 (0.3)
COVID-negative	230 (36.4)
Not tested but suspect to have been infected	6 (0.9)
Not tested and not suspect to have been infected	364 (57.6)
Not known	30 (4.8)
**District resided in (by no. of affected buildings**^c^	
High no. of affected buildings	223 (35.3)
Moderate no. of affected buildings	268 (42.4)
Low no. of affected building	141 (22.3)
**Risk perception**^d^	
Concerns over getting infected (1–10)	5.52 (2.67)
Concerns over their families getting infected (1–10)	6.19 (2.56)
Concerns over the lack of protective equipment (1–10)	6.00 (2.65)
Concerns over the continuous spread of the virus (1–10)	5.53 (2.36)

^a^ Students were excluded from subsequent analysis involving employment status.

^b^ Categorised into three groups according to the median monthly household income (HK$) in the “Population and Household Statistics Analysed by District Council District 2019” by the Census and Statistics Department of Hong Kong SAR.

^c^ Categorised according to number of residential buildings in which confirmed patients resided, and non-residential buildings (with 2 or more confirmed cases) had been visited by the confirmed cases (from 10/1/2020 to 09/10/2020), This information was retrieved on 12/1/2021 from the Coronavirus Disease (COVID-19) statistics in Hong Kong, provided by the Hong Kong Baptist University: https://beat-the-virus.hkbu.edu.hk/infographics/desktop_index.html)

^d^ The risk perception scores are presented as [Mean (Standard deviation)].

TCIM, Traditional, Complementary and Integrative Medicine.

Regarding risk perception, the respondents expressed moderate levels of concern about becoming infected (mean score = 5.52, standard deviation [SD] = 2.67), their family members becoming infected (mean score = 6.19, SD = 2.56), the lack of protective equipment during the initial outbreak (mean score = 6.00, SD = 2.65) and the continuous spread of the virus (mean score = 5.53, SD = 2.36).

### Pattern of TCIM use before and during COVID-19

Among the 632 respondents, 44.0% (n = 278) reported the use of at least one type of TCIM during the pandemic (Table 2). The most popular types of TCIM were vitamins or other dietary supplements (n = 160, 25.3%) and Chinese herbal medicine (n = 122, 19.3%). The respondents reported significantly less frequent use of TCIM during the pandemic as compared with the year before the pandemic started (44.0% vs 48.4%, p = 0.007), particularly with regard to Chinese herbal medicine (19.3% vs 28.6%, p<0.001), acupuncture (5.5% vs 9.7%, p<0.001) and massage or TuiNa (5.4% vs 10.4%, p<0.001).

**Table 2:** Pattern of TCIM use before and during COVID-19 (n = 632).

	All (%)	Before COVID-19 pandemic (%)^a^	During COVID-19 pandemic (%)	P^b^
**At least one type of TCIM**	342 (54.1)	306 (48.4)	278 (44.0)	**0.0069**
**Chinese herbal medicine**	196 (31.0)	181 (28.6)	122 (19.3)	**<0.001**
**Western herbal medicine**	45 (7.1)	39 (6.2)	32 (5.1)	0.17
**Vitamins or other dietary supplements**	191 (30.2)	169 (26.7)	160 (25.3)	0.27
**Acupuncture1**	65 (10.3)	61 (9.7)	35 (5.5)	**<0.001**
**Massage/TuiNa**	68 (10.8)	66 (10.4)	34 (5.4)	**<0.001**
**Aromatherapy**	33 (5.2)	31 (4.9)	26 (4.1)	0.18
**Yoga**	55 (8.7)	47 (7.4)	38 (6.0)	0.11
**Taichi/Qigong**	33 (5.2)	28 (4.4)	24 (3.8)	0.42
**Moxibustion/Tianjiu**	43 (6.8)	37 (5.9)	27 (4.3)	0.06

^a^ “Before COVID-19 pandemic” is defined as one year before December 2019.

^b^ McNemar test

TCIM, Traditional, Complementary and Integrative Medicine.

Among respondents who reported the use of TCIM during the pandemic, vitamin C (n = 69/278, 24.8%), vitamin B (n = 20/278, 7.2%), fish oil (n = 12/278, 4.3%) and probiotics (n = 11/278, 4.0%) were the most commonly used dietary supplements (S3 Table in S1 File). The most popular herbal products were Lingzhi (*Ganoderma Lucidum*) (n = 7/278, 2.5%), *Chrysanthemi Flos* (n = 5/278, 1.8%), *Isatidis Radix* (n = 5/278, 1.8%), and *Glycyrrhizae Radix Et Rhizoma* (n = 5/278, 1.8%).

### Reasons for TCIM use and the source of information

**Fig 1:**
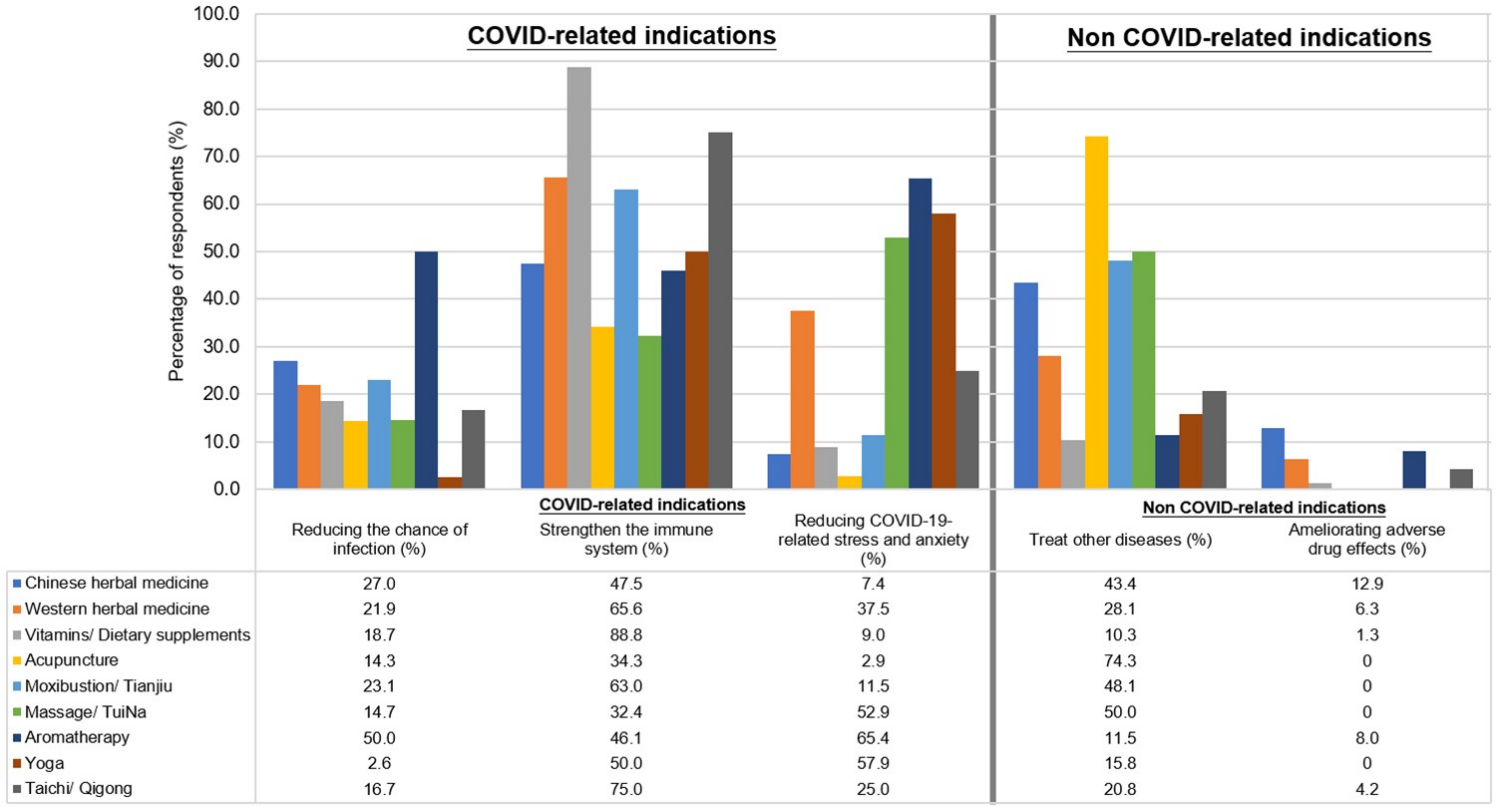
Reported indications for use of TCIM during COVID-19. TCIM, Traditional, Complementary and Integrative Medicine.

Among the participants who reported the use of specific TCIM, “strengthening the immune system” was the most frequently reported indication for using vitamins or other dietary supplements (n = 142/160, 88.8%), tai chi or qigong (n = 18/24, 75.0%) and western herbal medicines (n = 21/32, 65.6%) (Fig 1). Respondents also reported using TCIM, including aromatherapy (n = 17/26, 65.4%), yoga (n = 22/38, 57.9%) and massage or TuiNa (n = 18/34, 52.9%), to reduce stress or anxiety during COVID-19.

The respondents had obtained their information about TCIM mostly from friends or family members (n = 99/278, 35.6%), Chinese medicine practitioners (n = 88/278, 31.7%) and the Internet or social media (n = 83/278, 29.9%) (S4 Table in S1 File). A relatively small proportion of the respondents had obtained the information from physicians (n = 21/278, 7.6%) or pharmacists (n = 25/278, 9.0%).

### Predictors of TCIM use during the COVID-19 pandemic

**Fig 2:**
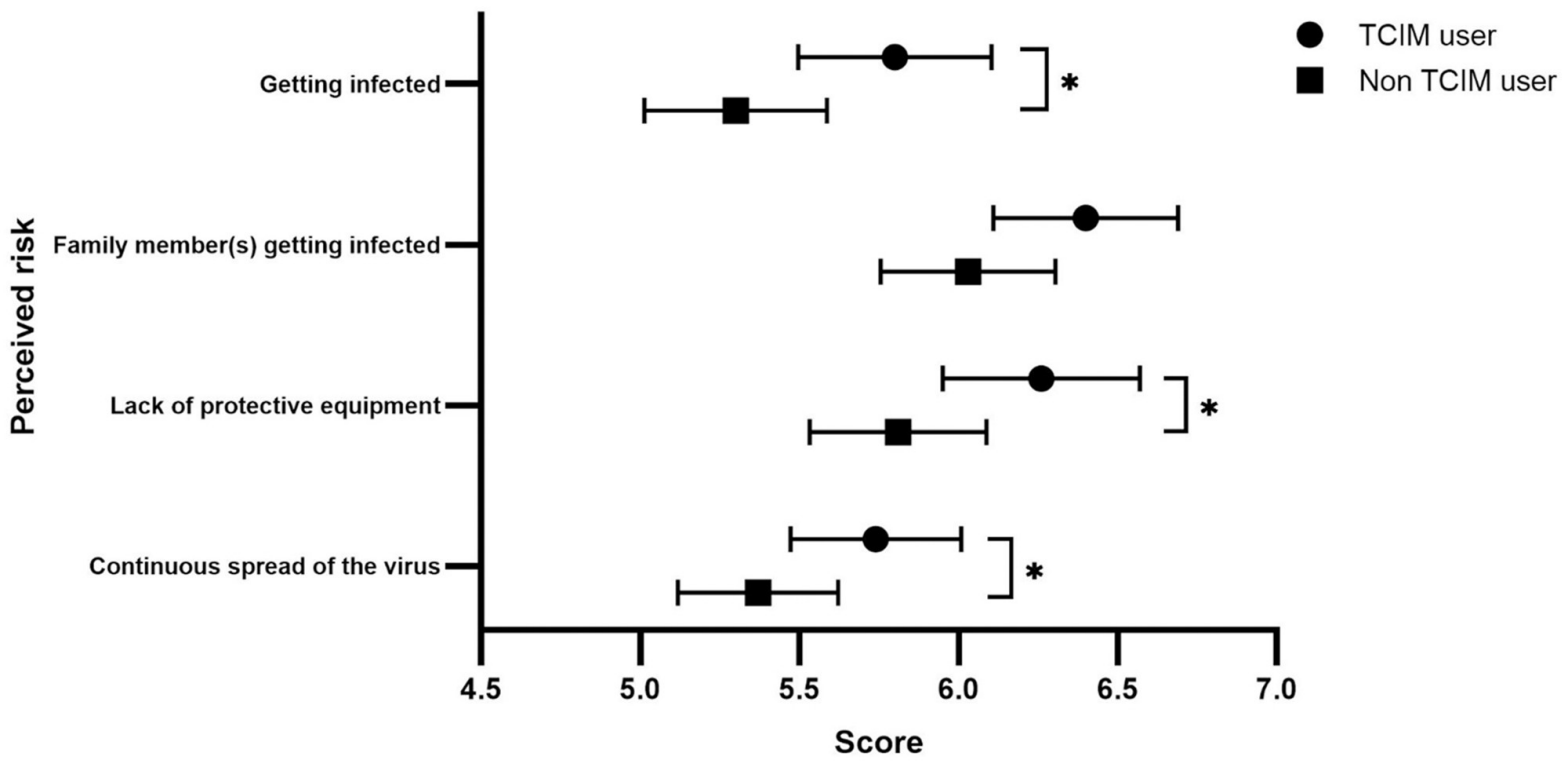
Risk perception score of COVID-19 among the respondents. ^a^ The risk perception scores were compared with Mann-Whitney U test. The scores are presented as mean with 95% confidence intervals. (*p<0.05) TCIM, Traditional, Complementary and Integrative Medicine.

The results of a univariate analysis indicated that the respondent’s reported gender, education level, age, religiosity, presence of chronic illnesses, severity of the outbreak in the residential area, risk perception and prior TCIM use were associated with TCIM use during the COVID-19 pandemic (Table 3). Regarding risk perception, there were significant differences (p<0.05) between TCIM users and non-users on all items except the level of concern about their family members becoming infected (Fig 2).

**Table 3:** Factors associated with use of TCIM during COVID-19 using logistic regression (n = 632).

	TCIM users during COVID-19 (n = 278)	Non-TCIM users during COVID-19 (n = 354)	Univariate		Multivariate^d^	
			Odds ratio (95% CI)	P	Odds ratio (95% CI)	P
**Demographic factors**						
**Gender**						
Male	82 (29.5)	151 (42.7)	Ref	**<0.001**	Ref	**<0.001**
Female	196 (70.5)	203 (57.3)	1.78 (1.28–2.49)		1.82 (1.29–2.59)	
**Age**						
18 to 35	100 (26.0)	171 (48.3)	Ref		Ref	
>35 to 55	118 (42.4)	116 (32.8)	1.74 (1.22–2.49)	**0.002**	1.77 (1.20–2.62)	**0.004**
>55	60 (21.6)	67 (18.9)	1.53 (1.00–2.35)	**0.05**	1.77 (1.04–3.02)	**0.03**
**Religion**						
Yes	134 (48.2)	118 (33.3)	1.86 (1.35–2.57)	**<0.001**	1.60 (1.14–2.25)	**0.007**
No	144 (51.8)	236 (66.7)	Ref		Ref	
**Socioeconomic factors**						
**Education level**						
Secondary school or below	41 (14.7)	75 (21.2)	Ref	**0.04**	Ref	**0.001**
Higher diploma, degree or above	237 (85.3)	279 (78.8)	1.55 (1.03–2.38)		2.21 (1.39–3.59)	
**Occupation**						
Employed	203 (73.0)	259 (73.2)	0.96 (0.64–1.45)	0.85		
Unemployed/retired/housewives	53 (19.1)	65 (18.4)	Ref			
**Family income**						
<10000	30 (10.8)	48 (13.6)	Ref	0.29		
>10000	248 (89.2)	306 (86.4)	1.30 (0.80–2.13)			
**District resided in (by median household income)**						
High-income districts	71 (25.5)	87 (24.6)	1.02 (0.68–1.54)	0.92		
Middle-income districts	108 (38.9)	143 (40.4)	0.95 (0.66–1.36)	0.76		
Low-income districts	99 (35.6)	124 (35.0)	Ref			
**Clinical factors**						
**Chronic illnesses**						
Yes	87 (31.3)	84 (23.7)	1.46 (1.03–2.08)	**0.03**	1.41 (0.93–2.14)	0.11
No	191 (68.7)	270 (76.3)	Ref		Ref	
**Chronic medication**						
Yes	40 (14.4)	46 (13.0)	0.70 (0.38–1.28)	0.25		
No	47 (16.9)	38 (10.7)	Ref			
**History of use TCIM**						
Yes	242 (87.1)	64 (18.1)	30.5 (19.8–48.0)	**<0.001**	30.7 (19.8–48.8)^a^	**<0.001**
No	36 (12.9)	290 (81.9)	Ref		Ref	
**Risk perception**						
**District resided in (by no. of affected buildings)**						
Low no. of affected buildings	51 (18.3)	90 (25.4)	Ref	**0.03**	Ref	**0.02**
Moderate to high no. of affected buildings	227 (81.7)	264 (74.6)	1.51 (1.03–2.24)		1.60 (1.07–2.42)	
**Risk perception score**^b^						
Concerns over getting infected (range 1 to 10)	5.80 (2.57)	5.30 (2.74)	1.07 (1.01–1.14)	**0.02**		
Concerns over their families getting infected (range 1 to 10)	6.40 (2.46)	6.03 (2.62)	1.06 (0.99–1.13)	0.8		
Concerns over the lack of protective equipment (range 1 to 10)	6.26 (2.62)	5.81 (2.66)	1.07 (1.01–1.14)	**0.04**		
Concerns over the continuous spread of the virus (range 1 to 10)	5.74 (2.27)	5.37 (2.41)	1.07 (1.00–1.13)	**0.05**		
Combined risk perception score (range 1 to 30)^c^	17.8 (6.35)	16.5 (6.49)	1.03 (1.01–1.06)	**0.01**	1.04 (1.01–1.07)	**0.008**

^a^ Adjusted for age and gender only.

^b^ The risk perception scores are presented as [Mean (Standard deviation)].

^c^ The combined risk perception score refers to the combination of “concerns over getting infected”, “concerns over the lack of protective equipment” and “concerns over the continuous spread of the virus” which were significant in the univariate analysis. They were combined as they were highly correlated with each other.

^d^ Variation inflation factor ranged from 1.01 to 1.10, suggesting absence of multicollinearity in the multiple regression models. Significance of the overall model (chi-square test of the difference between residuals): p < 0.001.

TCIM, Traditional, Complementary and Integrative Medicine.

In a multivariable analysis, respondents who reported using TCIM during the pandemic were more likely to be female (adjusted odds ratio [aOR] = 1.82, 95% confidence interval [CI] = 1.29–2.59) and to have a religious affiliation (aOR = 1.60, 95%CI = 1.14–2.25). The OR of using TCIM among middle-aged (aged 35 to 55 years: aOR = 1.77, 95%CI = 1.20–2.62) and older-aged adults (aged > 55 years: aOR = 1.77, 95%CI = 1.04–3.02) was higher than that among younger adults (aged 18 to 35 years). Furthermore, respondents who had attained a higher education level were twice as likely to use TCIM than were those who had attained a secondary school education or below (aOR = 2.21, 95%CI = 1.39–3.59). TCIM use before the initial COVID-19 outbreak was significantly associated with use during the pandemic (aOR = 30.7, 95%CI = 19.8–48.8).

In subgroup analyses, similar associations in the primary multivariable analysis on overall TCIM use were also identified in the subgroup analyses for vitamins or other dietary supplements users. Respondents with a higher family income (>HKD10,000: aOR = 2.44, 95%CI = 1.22–5.36) and chronic illnesses (aOR = 1.85, 95%CI = 1.17–2.95) were more likely to use vitamins or dietary supplements in addition to the factors identified above (S5 Table in S1 File). However, there were no significant differences between the users and non-users of Chinese herbal medicine except in the prior use of TCIM (S6 Table in S1 File).

## Discussion

We observed that more than 40% of the respondents in this study had used at least one form of TCIM during the pandemic. We also identified socio-demographics (female gender, older age, higher education attainment and having a religious affiliation), higher risk perception of COVID-19 and prior TCIM use, as factors associated with TCIM use during the pandemic.

To date, there are limited studies on the prevalence of TCIM use during the pandemic. Only a handful of existing studies evaluated TCIM use among infected patients [17–20], and even fewer were focused on TCIM as a preventive measure among the general public. Although the prevalence estimates of TCIM use in other countries during the pandemic are unknown, it is reasonable to speculate comparable trends of TCIM use in other East Asian countries, such as Taiwan, Japan and Korea, due to the deeply rooted culture and clearer institutional recognition of traditional medicine in most Asian societies [28]. Due to the important role of TCIM in the healthcare systems in these countries, future multinational effort and regional collaboration may shed light on the pattern of TCIM use in Asian societies for development of health policies and public education.

Interestingly, respondents in this study reported a lower rate of TCIM use during the pandemic, as compared to the pre-pandemic period. This is especially so for Chinese herbal medicine, acupuncture and massage or TuiNa. In Hong Kong, these modalities are often administered or prescribed by Chinese medicine practitioners. Under the quarantine policies and social distancing measures during the COVID-19 pandemic, people were likely discouraged from visiting complementary medicine practitioners. This is consistent with a US-based study that demonstrated a reduction in office-based visits for primary and specialty care during the COVID-19 pandemic [29]. As these modalities are often used as a complementary approach to the treatment of specific health conditions or for general health promotion [30, 31], a decrease in their usage may affect how patients manage their chronic conditions and health. Therefore, future studies can investigate whether similar reductions in TCIM use occurred in other regions and address the impact of decrease in TCIM use on the management of chronic diseases and health by the public during the COVID-19 pandemic.

Consistent with the findings of previous studies [21, 22], most survey respondents in this study used TCIM during the COVID-19 pandemic to strengthen the immune system. Vitamins were the most commonly used forms of TCIM. Vitamin C, D and E have known immunomodulatory effects [32], and some evidence suggests an association between vitamin D deficiency and increased incidence and severity of COVID-19, although the direct effect of supplementation is still under investigation [33]. In fact, emerging studies have been investigating the effectiveness of TCIM specifically for COVID-19. For example, a research group in Hong Kong has explored the effectiveness of targeted supplementation with specific probiotics on boosting immunity against the coronavirus [34]. Such robust scientific and clinical data to support the use of TCIM during a pandemic may help the public make informed decisions about TCIM approaches.

Unfortunately, we recognize that generating high-quality evidence on treatment or preventive approaches may not be feasible all the time, especially during a pandemic when timely responses are needed. In the case of TCIM, health authorities and professional bodies have been developing recommendations and guidelines largely based on expert advice and experience from previous pandemics [35, 36]. The findings inferred from our study may facilitate the development of such guidelines, as the values and preferences of the society are also an important factor to consider when developing relevant recommendations. Consequently, more resources should be allocated to TCIM modalities that are widely accepted and adopted by the public during the pandemic. Recently, the Hong Kong government launched the Special Chinese Medicine Programme to provide general Chinese medicine consultations to COVID-19 patients who remained in community treatment facilities and to offer rehabilitation services to those who were treated and discharged [37]. In the future, these services can help generate real-world data from pragmatic trials on the efficacy and safety of Chinese medicine use during the COVID-19 pandemic. This data may support the updates of the existing guidelines and inform treatment strategies for future pandemics.

In this study, most of the factors found to correlate with TCIM use were consistent with previously reported findings [21, 38, 39]. It is not surprising that individuals who have attained a higher education level may have a higher health literacy level [40], and hence may be more motivated to seek information to improve their health and maintain healthy behaviors. Older adults may have been more likely to engage in health-promoting behavior during the outbreak, given that worse outcomes of COVID-19 and higher rates of related mortality were more frequently reported among older populations [41]. However, the self-use of TCIM in these subgroups is not without safety concerns. For example, adverse and potentially harmful effects may occur due to inappropriate use. Particularly, herb-drug interactions might be a greater concern among older adults with more comorbidities [42]. This finding further emphasizes that health professionals and health authorities should provide accurate and useful information on the efficacy and safety of TCIM to the public.

In this study, a positive association of the risk perception of COVID-19 with TCIM use was also found. During a pandemic, increases in fear and perceived risk can increase engagement in preventive behaviors [43]. This was demonstrated clearly by the panic-buying of masks, hand sanitizers and other antiseptic materials in Hong Kong during the initial outbreak of COVID-19 [44]. People who expressed greater concern about being infected and the continuous spread of the virus were likely to actively seek different types of self-protective measures, including TCIM. A similar trend was observed during the MERS outbreak, during which TCIM users expressed greater concerns, higher levels of self-perceived danger and more frequent practices of hygiene measures [21]. The findings of these studies demonstrate that the use of TCIM may partially reflect the public’s health beliefs and confidence in the health system during a pandemic.

This study has a few limitations. First, as Hong Kong experienced a few pandemic waves that fluctuated in severity and extent [3], the TCIM use pattern might have differed between the periods. A follow-up survey could be conducted to study variations in TCIM use as the pandemic progressed. Second, the online survey was promoted through social media. There might be sampling bias as accessibility to the survey was restricted to those who actively used social media, for instance, older adults might have been precluded from participating because they were less likely to use these platforms [45]. Hence, the finding that older adults reported a higher rate of TCIM use in this study may require further validation in larger samples using alternative approaches (e.g., structured interviews or paper-based surveys in the community). However, these approaches were not feasible during the pandemic due to the social distancing rules. Finally, TCIM use was entirely self-reported in this study and was not verified using objective data. However, the respondents mostly used over-the-counter TCIM products that were self-purchased, products prescribed by private complementary medicine practitioners or self-administered treatments. Self-reporting, therefore, remains the most viable way to collect data regarding TCIM use, as this information would be largely excluded from medical charts or databases.

## Conclusion

In this cross sectional study, TCIM use was found to be common among the public in Hong Kong during the COVID-19 pandemic and was associated with well-documented sociodemographic factors in the literature. The respondents’ risk perception as a predictor of TCIM use may partially reflect the public’s behavioral response during a pandemic. Future studies can explore the health beliefs and motivations of TCIM users. While we acknowledge that vaccination, social distancing and basic hygiene remain the mainstay of controlling the pandemic, professional bodies of TCIM should take into consideration the evolving clinical evidence, experience from previous pandemics and the preferences in the society when they develop recommendations on TCIM use for COVID-19. Such recommendations should be disseminated to the public through the mass media to help the public make informed choices on the use of TCIM as preventive measures during the pandemic. Lastly, the collaboration among the key stakeholders (local health authorities, medical and TCIM professional bodies, and patient advocates) is crucial in establishing the long-term clinical and research infrastructure to guide TCIM use in future pandemics.

## Supporting information

S1 File (PDF)
