# Supplementary material for: A public survey of traditional, complementary and integrative medicine use during the COVID-19 outbreak in Hong Kong
Source: PLoS One. 2021 Jul 1;16(7):e0253890. doi: 10.1371/journal.pone.0253890 (PMC8248652; doi:10.1371/journal.pone.0253890)
Supplement: S1 File — (PDF) [file pone.0253890.s001.pdf]

S1 Table: The Checklist for Reporting Results of Internet E-Surveys (CHERRIES)

Item Category	Checklist Item	Details
**Design**	Describe survey design	This was an online cross-sectional study.
**IRB (Institutional Review Board) approval and informed consent process**	IRB approval	The study was approved by the Survey and Behavioural Research Ethics Committee of the Chinese University of Hong Kong (Reference no. SBRE-20-101).
	Informed consent	At the beginning of the survey, the study objectives were explained, and the participants were asked to provide informed consent before proceeding to the questions.
	Data protection	The questionnaire results were anonymous and were available only to the researchers involved in the analyses.
**Development and pre-testing**	Development and testing	The questionnaire was run and managed using Qualtrics XM (Qualtrics, Provo, UT), an online survey development platform and was reviewed by two researchers to assess the appropriateness and clarity of the questions. Pilot testing of the questionnaire was conducted on 10 individuals to ensure the feasibility of the procedure and the readability of the items for targeted respondents.
**Recruitment process and description of the sample having access to the questionnaire**	Open survey versus closed survey	This was an open survey that permitted participants to respond if they had the link to the questionnaire.
	Contact mode	The link of the survey was posted on publicly accessible social media platforms hosted by an academic institution. Eligible participants were invited to respond to the survey via social media platforms and they were encouraged to forward the link to other potential participants.
	Advertising the survey	Same as above
**Survey administration**	Web/E-mail	This was a web survey.
	Context	N/A
	Mandatory/voluntary	This was voluntary in nature.
	Incentives	N/A
	Time/Date	This study was conducted over a 6-week period from 2 November to 18 December 2020.
	Randomization of items or questionnaires	N/A
	Adaptive questioning	Adaptive questioning, in which subsequent questions were conditionally displayed based on responses to previous questions, was used to reduce the

		number and complexity of the survey. For instance, questions on reasons for using TCIM were displayed based on the types of TCIM used by the respondents.
	Number of items	The survey contained a total of 30 questions.
	Number of screens (pages)	The first section of the questionnaire contained nine questions (4 pages, 1-4 items per page). The second section (1 page, 6 items) contained six questions. The last section (2-4 pages, 1-7 items per page) contained fifteen questions.
	Completeness check	The respondents were required to complete mandatory questions before proceeding to the next pages.
	Review step	They were able to go backwards to review or change their responses before confirming that they had completed the survey.
**Response rates**	Unique site visitor	Each response with a unique IP address was counted as a valid response.
	View rate (Ratio of unique survey visitors/unique site visitors)	N/A
	Participation rate (Ratio of unique visitors who agreed to participate/unique first survey page visitors)	N/A
	Completion rate (Ratio of users who finished the survey/users who agreed to participate)	The completion rate of the survey was 88.1% (n=632/717).
**Preventing multiple entries from the same individual**	Cookies used	N/A
	IP check	Each response with a unique IP address was counted as valid.
	Log file analysis	N/A
	Registration	N/A
**Analysis**	Handling of incomplete questionnaires	Incomplete questionnaires were excluded from the statistical analyses.
	Questionnaires submitted with an atypical timestamp	N/A
	Statistical correction	N/A

Reference: Eysenbach G. Improving the quality of Web surveys: the Checklist for Reporting Results of Internet E-Surveys (CHERRIES). *Journal of medical Internet research.* 2004;6(3):e34.

S2 Table: Structure of the survey questionnaire

Section	No. of questions	Theme	Content coverage
**1**	9	Sociodemographic characteristics	• Age, gender, education level, religion, employment status, income level, residential areas, chronic diseases, and medication history
**2**	6	COVID status and risk perception	• Whether the respondents and their families had undergone COVID-testing or whether they doubt themselves getting infected if not tested<br>• Risk perception: A 11-point scale (0=not at all, 10=very much) was used in assessing the level of concern over:<br>o Respondents themselves getting infected during the initial outbreak<br>o Their families getting infected during the initial outbreak<br>o The lack of protective equipment<br>o The continuous spread of the virus
**3**	15	Pattern of TCIM use	• The use of TCIM before and during the pandemic<br>• If the participant has taken any herbal products, dietary supplements, or aromatherapy during the outbreak, the survey will proceed to further inquire about:<br>o The products they had taken or used<br>o The reasons for using a particular type of TCIM, such as reducing the chance of getting infected, strengthening the immune system, reduce COVID-related stress and anxiety, ameliorating side effects of drugs, or treating other diseases<br>o The sources of information

TCIM, Traditional, Complementary and Integrative Medicine.

S3 Table: List of most common products used during the COVID-19 pandemic

	**N=278 (%)**
**Vitamins/Dietary supplements**	
Vitamin C	69 (24.8)
Vitamin B	20 (7.2)
Fish Oil	12 (4.3)
Probiotics	11 (4.0)
Multivitamins	7 (2.5)
**Herbal**	
Lingzhi (*Ganoderma Lucidum*)	7 (2.5)
Chrysanthemi Flos	5 (1.8)
Isatidis Radix	5 (1.8)
Glycyrrhizae Radix Et Rhizoma	5 (1.8)
Yinqiao Jiedu Pian	4 (1.4)

S4 Table: Source of information of TCIM

	N=278 (%)
**Friends/family members**	99 (35.6)
**Chinese medicine practitioners**	88 (31.7)
**Internet/social media**	83 (29.9)
**Conventional media (newspaper, magazines, television commercials)**	35 (12.6)
**Pharmacists**	25 (9.0)
**Other healthcare professionals (nurses, physiotherapists, dietitians)**	24 (8.6)
**Physicians**	21 (7.6)
**Others**	17 (6.1)

TCIM, Traditional, Complementary and Integrative Medicine.

S5 Table: Factors associated with use of vitamins or other dietary supplements during COVID-19 using logistic regression (n=632)

	Users of vitamins or other dietary supplements during COVID-19 (n=160)	Non-users of vitamins or other dietary supplements during COVID-19 (n=472)	Univariate		Multivariate[d]	
			Odds ratio (95% CI)	*P*	Odds ratio (95% CI)	*P*
**Demographic factors**						
**Gender**						
Male	46 (28.8)	187 (39.6)	Ref	**0.01**	Ref	**0.006**
Female	114 (71.2)	285 (60.4)	1.62 (1.11-2.41)		1.77 (1.18-2.69)	
**Age**						
18 to 35	56 (35.0)	215 (45.5)	Ref		Ref	
>35 to 55	75 (46.9)	159 (33.7)	1.81 (1.21-2.72)	**0.004**	1.80 (1.16-2.79)	**0.009**
>55	29 (18.1)	98 (20.8)	1.14 (0.68-1.88)	0.62	1.38 (0.73-2.58)	0.31
**Religion**						
Yes	80 (50.0)	172 (36.4)	1.74 (1.21-2.51)	**0.003**	1.48 (1.01-2.19)	**0.04**
No	80 (50.0)	300 (63.6)	Ref		Ref	
**Socioeconomic factors**						
**Education level**						
Secondary school or below	18 (11.2)	98 (20.8)	Ref	**0.008**	Ref	**0.001**
Higher diploma, degree or above	142 (88.8)	374 (79.2)	2.07 (1.23-3.64)		2.65 (1.49-4.92)	
**Employment status**						
Employed	125 (78.1)	337 (71.4)	1.45 (0.90-2.42)	0.14		
Housewives/unemployed/retired	24 (15.0)	94 (19.9)	Ref			
**Family income**						
≤$10000	10 (6.2)	68 (14.4)	Ref	**0.008**	Ref	**0.02**
>$10000	150 (93.8)	404 (85.6)	2.52 (1.32-5.33)		2.44 (1.22-5.36)	
**District resided in (by median household income)**						
High-income districts	43 (26.9)	115 (24.4)	1.40 (0.87-2.25)	0.17		
Middle-income districts	70 (43.7)	181 (38.3)	1.45 (0.95-2.22)	0.09		
Low-income districts	47 (29.4)	176 (37.3)	Ref			
**Clinical factors**						
**Chronic illnesses**						
Yes	55 (34.4)	116 (24.6)	1.61 (1.09-2.36)	**0.02**	1.85 (1.17-2.95)	**0.009**
No	105 (65.6)	356 (75.4)	Ref		Ref	
**Chronic medication**						
Yes	24 (15.0)	62 (13.1)	0.67 (0.35-1.28)	0.23		
No	31 (19.4)	54 (11.4)	Ref			
**History of using TCIM**						
Yes	143 (89.4)	163 (34.5)	15.9 (9.57-28.2)	**<0.001**	15.4 (9.23-27.33)[a]	**<0.001**
No	17 (10.6)	309 (65.5)	Ref		Ref	

**Risk perception**						
**District resided in (by no. of affected buildings)**						
Low no. of affected buildings	26 (16.2)	115 (24.4)	Ref	**0.03**	Ref	**0.02**
Moderate to high no. of affected buildings	134 (83.8)	357 (75.6)	1.66 (1.05-2.70)		1.82 (1.13-3.03)	
**Risk perception score** [b]						
Concerns over getting infected (range 1 to 10)	6.04 (2.47)	5.34 (2.72)	1.11 (1.03-1.19)	**0.004**		
Concerns over their families getting infected (range 1 to 10)	6.60 (2.36)	6.05 (2.61)	1.09 (1.01-1.18)	**0.02**		
Concerns over the lack of protective equipment (range 1 to 10)	6.37 (2.53)	588 (2.68)	1.07 (1.00-1.15)	**0.04**		
Concerns over the continuous spread of the virus (range 1 to 10)	5.98 (2.14)	5.38 (2.41)	1.12 (1.00-1.14)	**0.006**		
**Combined risk perception score (range 1 to 40)** [c]	26.0 (7.94)	23.6 (8.7)	1.03 (1.01-1.06)	**0.003**	1.04 (1.01-1.06)	**0.004**

[a] Adjusted for age and gender only.

[b] The risk perception scores are presented as [Mean (Standard deviation)].

[c] The combined risk perception score refers to the combination of all four risk perception scores.

[d] Variation inflation factor ranged from 1.01 to 1.12, suggesting absence of multicollinearity in the multiple regression models. Significance of the overall model (chi-square test of the difference between residuals): $p < 0.001$.

TCIM, Traditional, Complementary and Integrative Medicine.

S6 Table: Factors associated with use of Chinese herbal medicine (CHM) during COVID-19 using logistic regression (n=632)

	CHM user during COVID-19 (n=122)	Non-CHM user during COVID-19 (n=510)	Univariate odds
**Demographic factors**			
**Gender**			
Male	39 (32.0)	194 (38.0)	Ref
Female	83 (68.0)	316 (62.0)	1.31 (0.86-2.01)
**Age**			
18 to 35	48 (39.4)	223 (43.7)	Ref
>35 to 55	52 (42.6)	182 (35.7)	1.33 (0.86-2.06)
>55	22 (18.0)	105 (20.6)	0.97 (0.55-1.68)
**Religion**			
Yes	56 (45.9)	196 (38.4)	1.36 (0.91-2.02)
No	66 (54.1)	314 (61.6)	Ref
**Socioeconomic factors**			
**Education level**			
Secondary school or below	18 (14.8)	98 (19.2)	Ref
Higher diploma, degree or above	104 (85.2)	412 (80.8)	1.37 (0.81-2.44)
**Employment status**			
Employed	94 (77.0)	368 (72.2)	1.52 (0.89-2.74)
Housewives/unemployed/retired	17 (13.9)	101 (19.8)	Ref
**Family income**			
≤$10000	17 (13.9)	61 (12.0)	Ref
>$10000	105 (86.1)	449 (88.0)	0.84 (0.48-1.54)
**District resided in (by median household income)**			
High-income districts	26 (21.4)	132 (25.9)	0.72 (0.42-1.21)
Middle-income districts	48 (39.3)	203 (39.8)	0.86 (0.55-1.36)
Low-income districts	48 (39.3)	175 (34.3)	Ref
**Clinical factors**			
**Chronic illnesses**			
Yes	37 (30.3)	134 (26.3)	1.22 (0.79-1.87)
No	85 (69.7)	376 (73.7)	Ref
**Chronic medication**			
Yes	15 (12.3)	71 (13.9)	0.60 (0.28-1.26)
No	22 (18.0)	63 (12.4)	Ref
**History of using TCIM**			
Yes	115 (94.3)	191 (37.5)	27.4 (13.5-66.0)[a]***
No	7 (5.7)	319 (62.5)	Ref
**Risk perception**			
**District resided in (by no. of affected buildings)** [b]			
Low no. of affected buildings	24 (19.7)	117 (22.9)	Ref
Moderate to high no. of affected buildings	98 (80.3)	393 (77.1)	1.22 (0.75-2.02)
**Risk perception** [b]			
Concerns over getting infected (range 1 to 10)	5.80 (2.83)	5.45 (2.64)	1.05 (0.97-1.13)
Concerns over their families getting infected (range 1 to 10)	6.47 (2.49)	6.13 (2.57)	1.06 (0.98-1.14)
Concerns over the lack of protective equipment (range 1 to 10)	6.21 (2.81)	5.95 (2.61)	1.04 (0.96-1.12)
Concerns over the continuous spread of the virus (range 1 to 10)	5.70 (2.41)	5.49 (2.34)	1.04 (0.96-1.13)

*** $p<0.001$

[a] Adjusted for age and gender only.

[b] The risk perception scores are presented as [Mean (Standard deviation)].
